# A Rare Case of Metastatic Urethral Squamous Cell Carcinoma Presenting with Paraneoplastic Sweet Syndrome and Treated with Pembrolizumab

**DOI:** 10.3390/curroncol32120683

**Published:** 2025-12-03

**Authors:** Dan-Thanh Christine Nguyen, Zineb Hamilou, Evelyne Bonnardeaux, Normand Blais, Manon de Vries-Brilland

**Affiliations:** 1 Department of Hematology-Oncology, Centre Hospitalier de l’Université de Montréal, Montréal, QC H2X 1R6, Canada; dan-thanh.christine.nguyen@umontreal.ca (D.-T.C.N.);; 2 Department of Dermatology, Centre Hospitalier de l’Université de Montréal, Montréal, QC H2X 1R6, Canada; 3 Department of Medical Oncology, Integrated Centers of Oncology (ICO) Paul Papin, 49055 Angers, France

**Keywords:** urothelial cancer, metastasis, immunotherapy, Sweet syndrome

## Abstract

Primary urethral cancer is an extremely rare malignancy, accounting for less than 1% of all cancers. Due to its rarity, evidence-based treatment recommendations are lacking. We report the case of a 44-year-old woman with metastatic squamous cell urethral carcinoma and paraneoplastic Sweet syndrome. The tumor was p16-positive with strong PD-L1 expression (CPS > 50%). Following surgery and adjuvant chemoradiotherapy, the patient developed hepatic and lymph node metastases. Pembrolizumab was initiated as first-line systemic therapy because of prior hematologic toxicity with cisplatin. After four cycles, complete radiologic remission of metastases and full resolution of the Sweet syndrome were achieved. This case highlights the potential benefit of immune checkpoint inhibitors in metastatic urethral SCC, particularly in p16-positive and PD-L1-high tumors, suggesting an inflamed and immunogenic microenvironment. To our knowledge, this is the first reported case of paraneoplastic Sweet syndrome successfully treated with pembrolizumab. These findings underscore the need for further investigation of immunotherapy in this rare and challenging malignancy.

## Simple Summary

Primary urethral cancer is an extremely rare cancer. Urethral squamous cell carcinoma is a type of urethral cancer. Due to its rarity, few treatments are available for metastatic urethral cancer. Immunotherapy has demonstrated its benefits in many other types of cancer. Our article presents a case of a 44-year-old woman with urethral squamous cell carcinoma. Following initial treatment with surgery and a combination of chemotherapy and radiotherapy, she developed hepatic and lymph nodes metastasis. She also presented a severe inflammatory skin condition caused by this cancer. Immunotherapy with Pembrolizumab was initiated. After four cycles of treatment, she achieved remission on follow-up scans, and her skin condition was also resolved. To our knowledge, this is the first reported case of metastatic urethral squamous cell carcinoma treated with immunotherapy. This study highlights the potential role of immunotherapy and the need for larger studies to evaluate this treatment.

## 1. Introduction

Primary urethral cancer is a rare malignancy, representing less than 1% of all cancers. The three most common histological subtypes are urothelial carcinoma, squamous cell carcinoma and adenocarcinoma. Squamous cell carcinoma represents approximately 12–35% of cases in men and 25–28% of cases in women [1]. Surgery remains the cornerstone in treating localized urothelial cancer. Multimodal treatment, such as neoadjuvant chemotherapy, followed by surgery or chemoradiotherapy is favored for advanced disease. Given the rarity of this disease, evidence-based treatment guidelines are limited concerning systemic therapy, especially for squamous cell carcinoma [1,2,3].

Immunotherapy has shown increasing promise in oncology. Checkpoint inhibitors restore anti-tumor immunity by blocking pathways that tumors use to evade the immune system [4]. They have become essential in treatment in a wide range of cancers, including squamous cell tumors such as head and neck squamous cell carcinoma, cervical cancer, lung cancer and non-squamous cell tumors [4,5,6,7]. In certain cancer types, validated biomarkers serve as predictors of immunotherapy efficacy, as p16-positive status [8] or the combined positive score (CPS) PD-L1 [9]. Unfortunately, no studies have yet demonstrated the effectiveness of immunotherapy in treating urethral squamous cell carcinoma.

We report a unique case of a patient with metastatic urethral squamous cell carcinoma who developed paraneoplastic Sweet syndrome and subsequently achieved a marked response to Pembrolizumab. Very few paraneoplastic syndromes have been reported with urethral cancer. Sweet syndrome, also known as acute febrile neutrophilic dermatosis, is a dermatologic condition characterized by the neutrophilic infiltration of the skin, resulting in nodules and erythematous-to-violaceous plaques. It is often accompanied by fever and leukocytosis. A total of 21% of cases are paraneoplastic and are mainly associated with hematologic neoplasms. They are rarely associated with solid tumors [10]. This is the first reported case of urethral squamous cell carcinoma treated with Pembrolizumab. Written informed consent from the patient was obtained.

## 2. Detailed Case Description

We report the case of a 44-year-old woman with a medical history of stage IIa Hodgkin lymphoma, in remission for 11 years following eight cycles of ABVD chemotherapy (doxorubicin, bleomycin, vinblastine, and dacarbazine). She also had a history of cervical precancerous lesions, treated with conization and a loop electrosurgical excision procedure (LEEP).

She initially presented with recurrent urinary tract infections and underwent cystoscopy, which identified a suspicious lesion originating from the bladder neck invading the totality of the urethra. Biopsy of the lesion revealed a high-grade carcinoma with squamous differentiation and subepithelial invasion. Molecular testing was positive for expression.

Initial Pelvic MRI demonstrated a lesion of 9 mm × 55 mm of the urethra with intra vesical enhancement. The case was discussed by a multidisciplinary tumor board. One month later, the patient underwent radical cystectomy by pelvic exenteration, uretrectomy, and bilateral pelvic lymphadenectomy with Indiana pouch reconstruction. Histopathology confirmed a squamous cell carcinoma, pT2N0, with an extensive superficial papillomatous component exhibiting a basaloid condylomatous pattern, associated with high-risk HPV infection. The tumor extended along the urethral tract, bladder neck, and partially invaded the bladder wall, sparing the bladder dome. Surgical margins were negative. Lymphovascular invasion was identified, but no lymph node metastases were found. PD-L1 assay was performed by immunohistochemistry. Next-generation sequencing (NGS) was not obtained. PD-L1 expression was positive, with a combined positive score (CPS) exceeding 50% and a tumor cell score estimated above 80%. The case was rediscussed by the multidisciplinary tumor board. She underwent additional bilateral inguinal lymphadenectomy three months after her initial surgery. Histopathological examination revealed metastasis in the left superficial lymph node. A PET scan performed to investigate post-operative fever showed no evidence of further metastasis.

Three months after her bilateral inguinal lymphadenectomy, the patient began adjuvant concomitant chemoradiotherapy. A five-week course of cisplatin at 40 mg/m^2^ per week was prescribed alongside radiotherapy targeting the inguinal region. However, after the first cycle, she developed grade 4 febrile neutropenia. The second dose was delayed by one week and administered with G-CSF support, but she subsequently developed grade 3 thrombocytopenia. Cisplatin was discontinued thereafter. Post-treatment, the patient experienced persistent grade 1 thrombocytopenia. She completed a total of 18 fractions of radiotherapy (3600 cGy) delivered bilaterally to the groin, followed by 7 additional fractions (1400 cGy) to the bilateral inguinal regions, and 2 supplementary fractions (400 cGy) to the left groin.

Six months after her adjuvant therapy, a routine CT scan revealed a hypodense lesion at the junction of liver segments V and VI. Subsequent liver MRI suggested a 9 mm hepatic metastasis in the same area.

**Figure 1:**
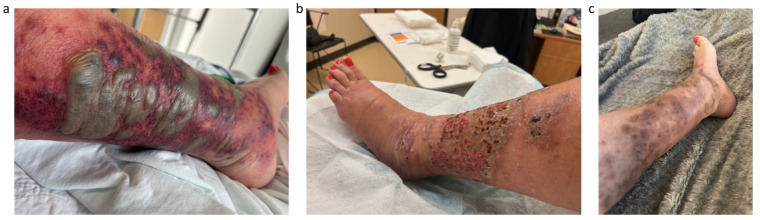
Evolution of paraneoplasic Sweet Syndrome—(**a**) initial presentation before initiation of prednisone, (**b**) clinical appearance at the start of Pembrolizumab, (**c**) clinical appearance after 3 months of Pembrolizumab.

Two months later, the patient was hospitalized for fever, bullous erythema on the left leg with scattered violaceous plaques (Figure 1a) and severe pancytopenia. She underwent evaluation by dermatology, infectious disease, and hematology specialists.

Liver biopsy during her hospitalization confirmed metastatic squamous cell carcinoma. A bone marrow biopsy revealed normocellular marrow with mild monoclonal plasmacytosis for the patient’s age, suggestive of monoclonal gammopathy of undetermined significance (MGUS) that could not explain the patient’s presentation. No evidence of myeloproliferative, myelodysplastic, or lymphoproliferative disorders were found.

Her skin condition was initially treated empirically as cellulitis. She received antibiotics, including cefazolin, for 4 days. Her condition was not improving, and treatment was changed to vancomycine, piperacillin tazobactam and ciprofloxacine.

A skin biopsy from the left leg showed marked edema of the papillary dermis and a dense dermal inflammatory infiltrate rich in neutrophils and histiocytes, raising the possibility of histiocytoid Sweet syndrome. Antibiotics were stopped after a total of 8 days and the patient was started on prednisone, 50 mg daily for 5 days, followed by a progressive taper over 10 weeks. Dermatological evaluation reported a partial clinical response following completion of treatment, with residual erythematous plaques and areas of post-inflammatory hyperpigmentation (Figure 1b).

Pembrolizumab at 2 mg/kg every 3 weeks was initiated one month after her hospitalization. After discussion with the patient, Pembrolizumab was chosen over alternative chemotherapy options such as capecitabine, due to her prior cytopenia related to cisplatin.

**Figure 2:**
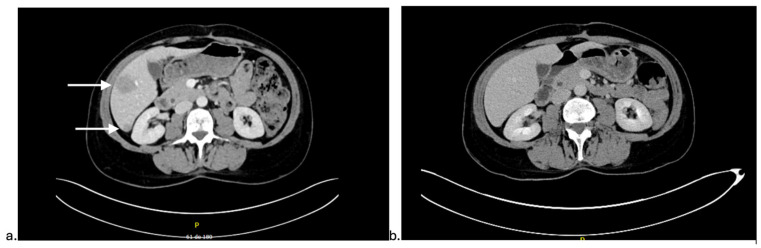
Radiological evolution of metastatic urethral squamous cell carcinoma at the start of Pembrolizumab (**a**) and after 3 months of Pembrolizumab (**b**).

Abdominopelvic and thoracic CT imaging performed before treatment initiation showed progression of the hepatic metastasis at the junction of segments V and VI from 9 mm to 28 mm, along with new hepatic lesions measuring 12 mm (segment VI), 6 mm (segments VI and VII), 15 mm (segment II), and 8 mm (segment IV). Additionally, a new 9 mm metastasis was identified in an interaortocaval and infrarenal lymph node (Figure 2a).

After 3 months (four doses) of Pembrolizumab, on dermatology follow-up, the Sweet syndrome had resolved, leaving post-inflammatory hyperpigmentation on the left leg (Figure 1c). Her ECOG performance status also improved from 2 to 0, and she reported increased appetite and significant weight gain. A routine CT scan demonstrated complete resolution of the hepatic lesions and the interaortocaval lymph node metastasis (Figure 2b).

## 3. Discussion

We reviewed the management of urethral cancer. For localized disease, complete tumor resection remains the primary treatment approach. For locally advanced disease, a series of 124 patients published by Gakis et al. supports multimodal therapy consisting of neoadjuvant chemoradiotherapy followed by surgery [1,2,11]. Metastatic urethral cancer is generally treated with systemic chemotherapy regimens adapted from those used in other squamous cell or urothelial carcinomas. For example, combinations such as 5-fluorouracil and cisplatin have been employed in squamous cell carcinoma of the urethra, though no consensus guidelines currently exist [12,13]. A retrospective study by Wenzel et al. examining metastatic urethral cancer reported improved survival following chemotherapy in most histologies, except for squamous cell carcinoma, highlighting an unmet need for further research in this challenging subset [14].

As mentioned earlier, immunotherapy, such as anti-PD1 therapy, has proved its benefits in many tumor types. The programmed death-1 (PD-1) receptor present on immune cells binds to its ligand PD-L1, which is overexpressed on tumor cells. This interaction suppresses T-cell proliferation and triggers apoptosis, limiting the immune system’s ability to recognize and eliminate tumor cells. Malignant cells leverage this checkpoint pathway and the surrounding immunosuppressive microenvironment to escape immune surveillance. Monoclonal anti-bodies targeting PD1, such as Pembrolizumab, are recognized as effective treatment for many other squamous cell tumor types by restoring anti-tumor immunity [1].

A retrospective study in a small cohort of patients treated with immunotherapy for oropharynx squamous cell carcinoma suggested that p16-positive status could suggest an association between p16 positivity and favorable outcomes (HR = 7.67) [8]. Supporting this, a pooled analysis published by Wang et al. suggested that HPV infection enhances T-cell infiltration and contributes to the development of an inflamed tumor microenvironment, which results in PD-L1 expression [15].

Our patient had a CPS exceeding 50% and positive p16 expression. Given her inability to tolerate additional platinum-based chemotherapy due to cytopenia, we decided to treat her with Pembrolizumab. A case report published in 2022 by Hongsong described a patient with urethral squamous cell carcinoma and inguinal metastasis who received four cycles of paclitaxel and carboplatin combined with toripalimab, a PD-1-blocking monoclonal antibody, every three weeks. The patient achieved a complete response after receiving the combination of chemotherapy and immunotherapy with no recurrence after 20 months of follow-up [16]. In our case, the patient showed a complete radiological response of her metastases after only four doses of Pembrolizumab.

Paraneoplastic Sweet syndrome presents as erythematous plaques or nodules with fever and neutrophilia. It is histologically characterized by neutrophilic infiltrate within the dermis, often accompanied by papillary dermal edema. It can be idiopathic or associated with malignancies or medications. When linked to malignancy, hematologic diseases account for approximately 85% of cases, including acute myeloblastic leukemia and myelodysplastic syndrome [10]. Less commonly, it is associated with solid tumors such as genitourinary, breast and gastrointestinal carcinomas [17].

Treatment of Sweet Syndrome typically involves systemic corticosteroids, although management of the underlying malignancy remains the cornerstone of therapy [10]. In fact, Sweet Syndrome can be resolved simply by treating the underlying malignancy. If corticosteroids are initiated, most patients respond to prednisone 0.5–1 mg/kg/day. Biologic agents such as Anakinra and Tocilizumab have been used for refractory cases. [18]. A small retrospective study published in 2022 reported that, in malignancy-induced Sweet Syndrome, lesions that did not fully resolve with corticosteroids improved after initiation of cancer-directed therapy [19]. However, guidelines are limited in management of malignancy-induced Sweet Syndrome due to its rarity.

In our case, Sweet syndrome showed a partial response to corticosteroids. Combination with Pembrolizumab led to its complete clinical resolution. Interestingly, several case reports have described Sweet syndrome to be induced by immunotherapy agents, including Pembrolizumab [20,21,22]. In contrast, Sweet syndrome in our patient was paraneoplastic, and Pembrolizumab did not exacerbate cutaneous symptoms but led to their resolution. To our knowledge, this represents the first reported case of paraneoplastic Sweet syndrome successfully treated with a checkpoint inhibitor, as well as a rare case of complete response of urethral squamous cell carcinoma to immunotherapy.

## 4. Conclusions

In conclusion, we report the first case of metastatic squamous cell urethral carcinoma, with a CPS greater than 50% and positive p16 status, complicated by paraneoplastic Sweet syndrome. This is the first case of metastatic squamous cell urethral carcinoma that has demonstrated a complete radiological response of hepatic and lymph node metastases, as well as a full clinical resolution of paraneoplastic Sweet syndrome, after four cycles of Pembrolizumab. This case underscores the challenges in treating metastatic squamous cell urethral cancer and highlights the potential role of immunotherapy. Further studies involving larger patient cohorts are needed to better evaluate the efficacy of immunotherapy in this population.

## Data Availability

The data in this study is unavailable due to privacy of the patient.
